# Development of β-Carotene Rich Maize Hybrids through Marker-Assisted Introgression of *β-carotene hydroxylase* Allele

**DOI:** 10.1371/journal.pone.0113583

**Published:** 2014-12-08

**Authors:** Vignesh Muthusamy, Firoz Hossain, Nepolean Thirunavukkarasu, Mukesh Choudhary, Supradip Saha, Jayant S. Bhat, Boddupalli M. Prasanna, Hari S. Gupta

**Affiliations:** 1 Division of Genetics, ICAR-Indian Agricultural Research Institute, New Delhi, India; 2 Division of Agricultural Chemicals, ICAR-Indian Agricultural Research Institute, New Delhi, India; 3 Regional Research Centre, ICAR-Indian Agricultural Research Institute, Dharwad, Karnataka, India; National Institute of Plant Genome Research (NIPGR), India

## Abstract

Development of vitamin A-rich cereals can help in alleviating the widespread problem of vitamin A deficiency. We report here significant enhancement of kernel β-carotene in elite maize genotypes through accelerated marker-assisted backcross breeding. A favourable allele (543 bp) of the *β-carotene hydroxylase* (*crtRB1*) gene was introgressed in the seven elite inbred parents, which were low (1.4 µg/g) in kernel β-carotene, by using a *crtRB1*-specific DNA marker for foreground selection. About 90% of the recurrent parent genome was recovered in the selected progenies within two backcross generations. Concentration of β-carotene among the *crtRB1-*introgressed inbreds varied from 8.6 to 17.5 µg/g - a maximum increase up to 12.6-fold over recurrent parent. The reconstituted hybrids developed from improved parental inbreds also showed enhanced kernel β-carotene as high as 21.7 µg/g, compared to 2.6 µg/g in the original hybrid. The reconstituted hybrids evaluated at two locations possessed similar grain yield to that of original hybrids. These β-carotene enriched high yielding hybrids can be effectively utilized in the maize biofortification programs across the globe.

## Introduction

Micronutrient malnutrition, mainly due to iron, zinc, and vitamin A deficiencies, has become one of the major health problems in the developing world [1]. Vitamin A deficiency (VAD) results in visual impairment and higher morbidity as well as mortality in at least 190 million pre-school children and 19 million pregnant women, mostly in Africa and South Asia [2]. It also causes low resistance to infectious diseases and accounts for about 70% of childhood deaths across the world [3]. The deficiency is particularly prevalent in rural populations in the developing countries, where staple diets are mostly deficient in micronutrients [4]. Although many strategies including supplementation, dietary diversification and fortification of foods have been deployed to overcome VAD, biofortification involving crop varieties that are rich in micronutrients promises to be a cost-effective and sustainable approach, which provides consumers with essential micronutrients in their natural form [5].

Maize is consumed by more than a billion people in sub-Saharan Africa, Latin America and in many countries in Asia [6], [7]. It has been targeted for biofortification of many nutrients for decades, and the efforts have largely been successful [8]–[10]. Maize with yellow kernels exhibits tremendous natural variation in kernel carotenoids having both provitamin A (α-carotene, β-carotene and β-cryptoxanthin) and non-provitamin A (lutein and zeaxanthin) components, and holds promise for β-carotene biofortification [11]. The challenge, however, lies in large-scale phenotyping for kernel carotenoids and methods such as high performance liquid chromatography (HPLC) are time-consuming as well as expensive. Moreover, kernel colour is not a reliable indicator of β-carotene concentration [12]; therefore, breeding programs involving marker-assisted selection (MAS) can be of immense use. MAS is a highly efficient breeding method as it allows precise selection of the target gene, thereby shortening the breeding cycle [13], [14]. It is the most effective way of transferring specific genes to otherwise an agronomically superior cultivar [15], [16]. In maize, marker-assisted backcross breeding (MABB) for nutritional quality has been used with success over a decade for developing genotypes with improved endosperm quality [6], [9], [17], [18] and reduced anti-nutritional factors [19].

The carotenoid biosynthesis pathway has been well characterised in maize. Among the genes involved in the pathway, *phytoene synthase 1* (*PSY1* or *Y1*) plays pivotal role by condensing two geranyl geranyl pyrophosphate molecules into a single molecule of phytoene [11]. Plants with *Y1* gene produce carotenoids, which imparts colour to the kernel in maize. The first branching point of the pathway is the cyclization of lycopene: the *lycopene epsilon cyclase* (*lcyE*) gene, in association with other genes, converts more lycopene to the β, ε branch, which produces more α-carotene and lutein. However, the naturally existing mutant alleles of *lcyE* divert more lycopene to the β, β branch, which produces β-carotene, β-cryptoxanthin, and zeaxanthin [12]. Although the favourable *lcyE* allele increases the proportion of β-carotene in the pathway, *β-carotene hydroxylase* (*crtRB1*) hydroxylates large amounts of that β-carotene to produce β-cryptoxanthin (which has provitamin A activity only half that of β-carotene) and zeaxanthin (which has no provitamin A activity at all) [20]. Therefore, blocking the hydroxylation can potentially increase the level of β-carotene relative to those of β-cryptoxanthin and the downstream zeaxanthin. Natural genetic variation in *crtRB1* gene has been reported by Yan et al. 2010 [21], and large-scale validation experiments indicate that one favourable allele, namely *crtRB1* 3′TE, alone is responsible for effecting 2 to 10-fold increase in kernel β-carotene concentration in maize [22]. Co-dominant marker for the 3′TE region of the *crtRB1* gene was identified using polymerase chain reaction (PCR) and would help in rapidly improving β-carotene concentration in maize kernels through MAS [21], [23]. Considering the importance of provitamin A biofortification and the potential of *crtRB1* gene in increasing the level of β-carotene in maize kernels, the present investigation was undertaken to introgress the favourable allele in elite inbred parents of agronomically superior commercial maize hybrids through MABB. The inbreds thus generated were used to reconstitute new hybrids that were evaluated for kernel β-carotene as well as agronomic performance to develop β-carotene-rich high yielding maize hybrids.

## Materials and Methods

### Plant materials

The experimental materials consisted of seven elite maize inbreds: VQL1, VQL2, V335, V345, HKI1105, HKI323, and HKI161 [a derivative of CML161- a Quality Protein Maize (QPM) inbred developed at CIMMYT, Mexico having wider adaptability and has been used as parent in hybrid program globally] (Table 1). Of these inbreds, VQL1, VQL2, and HKI161 are QPM genotypes with high tryptophan. The seven inbreds are the parents of four widely adapted, high yielding commercial maize hybrids in India, two extra-early-maturing [Vivek QPM-9 (VQL1×VQL2) and Vivek Hybrid-27 (V335×V345)] and two of medium duration [HM-4 (HKI1105×HKI323) and HM-8 (HKI1105×HKI161)]. However, all the four hybrids and their seven inbred parents have low levels of kernel β-carotene; hence were targeted for β-carotene enhancement. High β-carotene inbreds developed under CIMMYT-HarvestPlus program, served as the donors for introgression of the target gene into the parental lines of the hybrids (Table 1).

**Table 1:** Pedigree details of recurrent and donor parents used in the study.

Parents	Pedigree	Source
Recurrent parents		
VQL1	(CM 212×CML 189) BC3P1-∶b∶b∶b-#	VPKAS, Almora, India
VQL2	(CM 145×CML 170)BC3P1∶b∶b∶- ##	VPKAS, Almora, India
V335	(TZI-25 F-##-∶b-∶-4-1-∶b∶b-∶-14-###-∶b-#-∶b-##)	VPKAS, Almora, India
V345	BIO-45010 OP, F∶-2-1-8-5-5-∶B-#-∶B-##	VPKAS, Almora, India
HKI1105	Cargil 633	CCS-HAU, Uchani, India
HKI323	CIMMYT Pool 28	CCS-HAU, Uchani, India
HKI161	CML161	CCS-HAU, Uchani, India
**Donor parents**		
HP465-43	(KUI carotenoid syn-FS25-3-2-B-B-B/(KU1409/DE3/KU1409)S2-18-2-B)-B-2-3	CIMMYT-HarvestPlus
HP465-41	(KUI carotenoid syn-FS25-3-2-B-B-B/(KU1409/DE3/KU1409)S2-18-2-B)-B-2-1	CIMMYT-HarvestPlus
HP465-30	(KUI carotenoid syn-FS17-3-2-B-B-B/(KU1409/DE3/KU1409)S2-18-2-B)-B-1-5	CIMMYT-HarvestPlus
HP465-35	(KUI carotenoid syn-FS17-3-2-B-B-B/(KU1409/DE3/KU1409)S2-18-2-B)-B-3-1	CIMMYT-HarvestPlus
HP467-6	(KUI carotenoid syn-FS11-1-1-B-B-B/(KU1409/DE3/KU1409)S2-18-2-B)-B-2-4	CIMMYT-HarvestPlus
HP467-4	(KUI carotenoid syn-FS11-1-1-B-B-B/(KU1409/DE3/KU1409)S2-18-2-B)-B-2-2	CIMMYT-HarvestPlus
HP467-13	(KUI carotenoid syn-FS11-1-1-B-B-B/(KU1409/DE3/KU1409)S2-18-2-B)-B-3-1	CIMMYT-HarvestPlus

### Target gene(s) for introgression

**Figure 1:**
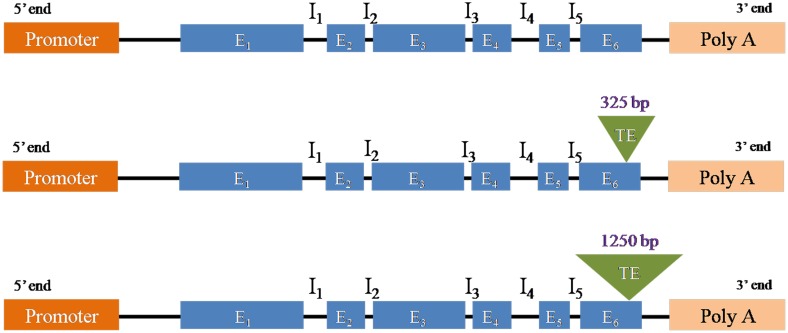
Structure of the alleles of *crtRB1* gene causing variation in β-carotene concentration of maize. E: Exon; I: Intron; TE: Transposable element. **A**: No TE insertion at 6^th^ Exon causing favourable *allele 1* (543 bp amplicon); **B**: 325 bp TE insertion at 6^th^ Exon causing unfavourable *allele 2* (296+875 bp amplicon); **C**: 1250 bp TE insertion at 6^th^ Exon causing unfavourable *allele 3* (296+1221+1880 bp amplicon).

The enzyme encoded by the *crtRB1* gene causes the hydroxylation of β-carotene in to non-provitamin A carotenoids [21]. The 3′TE (transposable element) polymorphism of the gene that spans the 6^th^ exon and the 3′-UTR (untranslated region) generates three alleles, namely *allele 1* (543 bp; without TE insertion), *allele 2* (296 bp+875 bp; with 325 bp TE insertion), and *allele 3* (296 bp+1221 bp+1880 bp; with 1250 bp TE insertion), that were associated with altering β-carotene accumulation [21] (Fig. 1). *Allele 1* of the *crtRB1* gene (hereafter *allele 1*) is favourable and increases the level of β-carotene, whereas *allele 2* and *allele 3* cause unfavourable effects [21]. Thus, *allele 1* was targeted for introgression. In addition, three of the seven recurrent parents are QPM genotypes, foreground selection using a gene based SSR marker (*umc1066,* present in the first exon of the gene) was carried out to retain *opaque2* (*o2*) allele [24].

### DNA isolation and polymerase chain reaction

Genomic DNA was isolated from 3-week-old seedlings using the standard CTAB procedure [25]. Polymerase chain reaction for the marker specific to the *crtRB1* 3′TE was performed using the following set of primers: *crtRB1*-3′TE-F: ACACCACATGGACAAGTTCG, *crtRB1*-3′TE-R1: ACACTCTGGCCCATGAACAC, and *crtRB1*-3′TE-R2: ACAGCAATACAGGGGACCAG. *For the PCR cycle, the procedure given by Yan et al. (2010) was followed [21]. Primers F and R2 amplify the intact crtRB1 3′TE region and produce a single amplicon (allele 1), whereas primer R1, which is specific to the TE insertion, amplifies the insertion region within the crtRB1 3′TE gene and generates more than one fragment, allele 2 and allele 3. The amplified fragments were resolved using agarose gel electrophoresis (1.5% agarose) and scored for the presence of allele polymorphism [21].*

The reaction for SSR markers was carried out in 10 µl reaction mixture containing 2 µl of 20 ng/µl genomic DNA as the template, 2 mM MgCl_2_, 1 mM dNTPs, 2 µM of the primer pair (forward and reverse), and 1.5 U Taq Polymerase (GeNei, Mumbai, India), and PCR amplification (Bio-Rad, California, USA) was carried out by a ‘touch-down’ procedure. The first step had 12 cycles: denaturation at 94°C for 30 s, annealing at 63°C for 30 s (annealing temperature was reduced subsequently by 0.5°C per cycle), and extension at 72°C for 45 s. The second step was set for 35 cycles: denaturation at 94°C for 30 s, annealing at 60°C for 30 s, and extension at 72°C for 45 s. The final extension was carried out at 72°C for 7 min. The amplicons were resolved using 3.5% superfine resolution (Amresco, USA) agarose gel electrophoresis and documented using a gel documentation system (AlphaInnotech, California, USA).

### Marker-assisted backcross breeding (MABB)

**Figure 2:**
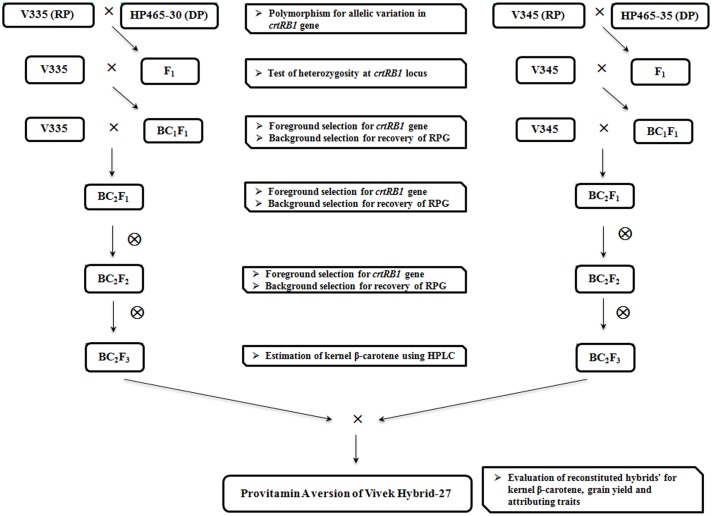
Marker-assisted backcross breeding scheme adapted for the introgression of *allele 1* of the *crtRB1* 3′TE gene in to elite parent (V335 and V345) of the maize hybrid Vivek Hybrid-27 (RP: Recurrent Parent; DP: Donor Parent).

The MABB scheme followed in the present study is presented in Fig. 2. Plant×plant crosses were made between recurrent parents (as females) and donors (as males) in 2010 during rainy season (July–October) at IARI Experimental Farm, New Delhi (28°08′N, 77°12′E, 229 MSL). The F_1_s of the seven crosses were grown at Winter Nursery Centre (WNC), Hyderabad (17°19′N, 78°24′E, 542.6 MSL), during winter season (December-April) in 2010/11. Heterozygosity of the F_1_s was tested using gene-specific marker; the true F_1_s were used as males and backcrossed to their respective recurrent parents. BC_1_F_1_ progenies were grown in Delhi during rainy season in 2011, and foreground selection was carried out using gene-specific marker(s). Heterozygous plants with high recovery of the recurrent parent genome (RPG) were further backcrossed with the respective recurrent parents to raise BC_2_F_1_ population. The selected heterozygous plants were selfed to generate BC_2_F_2_ plants. All seven crosses except VQL2×HP465-41 were generated by this procedure. BC_1_F_1_ could not be generated in winter season of 2010/11 for VQL2-based crosses, owing to the non-synchrony of flowering; however, the crosses were generated in rainy season of 2011 and the BC_1_F_2_ population was raised in rainy season of 2012. The selected plants in each crosses were selfed to generate BC_2_F_3_ progenies (BC_1_F_3_ in case of VQL2-based crosses), with a view to multiply the seeds of improved inbreds and to generate crosses among them to reconstitute the hybrids.

#### a) Marker-assisted foreground selection

Foreground selection was employed in BC_1_F_1_, BC_2_F_1_, and BC_2_F_2_ generations (BC_1_F_2_ in case of VQL2-based crosses) using a marker specific to *crtRB1*. Heterozygous plants (*allele 1*/*allele 3*) were selected in the BC_1_F_1_ and BC_2_F_1_ generations and homozygotes (*allele 1*/*allele 1*) were selected in the BC_2_F_2_/BC_1_F_2_ generation. The chi-square test was performed using the standard procedure for testing the goodness of fit of the observed segregation pattern at the *crtRB1* locus in each of the generations. For the QPM inbreds, namely VQL1, VQL2, and HKI161, foreground selection for *o2* allele was also carried out in *crtRB1*-positive lines using *umc1066*.

#### b) Marker-assisted background selection

A set of 200 genome-wide SSRs covering all the 10 chromosomes of maize was used for identifying polymorphic markers between the respective recurrent and donor parents. The sequences of the SSR primers were adapted from the maize genome database (www.maizegdb.org) and custom-synthesized (SigmaTech., USA). These polymorphic SSR markers were employed in BC_1_F_1_ and BC_2_F_1_ generations to recover the RPG.

### Evaluation of introgressed inbreds for kernel β-carotene

BC_2_F_3_ seeds (BC_1_F_3_ for VQL2-based progenies) from randomly selected three homozygous progenies were used for initial analyses to study the effect of *crtRB1* on accumulation of kernel β-carotene. Later, 13 selected BC_2_F_4_ (BC_1_F_4_ for VQL2-based progenies) were evaluated along with the respective recurrent and donor parents during rainy season in 2013 at IARI Experimental Farm, New Delhi. The trial was conducted using randomised complete block design (RCBD) with two replications, each having two rows (each of 3 m length) with plant-to-plant spacing of 20 cm and row-to-row spacing of 75 cm. To estimate kernel β-carotene, three cobs in each of the genotypes per replication were self-pollinated to avoid possible xenia effects.

### Reconstitution of hybrids

The 13 selected improved progenies of the seven inbreds were used in crossing program to reconstitute the F_1_ hybrids during winter season of 2012/13 at WNC, Hyderabad. The reconstituted experimental hybrids along with their corresponding original hybrids were evaluated for their agronomic performance at two diverse maize growing zones of the country viz. IARI Experimental Farm, New Delhi in Northern India and IARI Regional Research Centre, Dharwad (15°21′N, 75°05′E, 750 MSL), Karnataka in Southern India, during rainy season, 2013. Trials were evaluated in two replications having same row and plant specifications as followed for inbred trial. Grain yield and its attributing traits (days to 50% anthesis, days to 50% silking, plant height, cob height, cob length, cob girth, number of kernel rows and 100 kernel weight) were recorded in both the locations. Since kernel carotenoids in maize is stable over environments [26], [27], concentration of kernel β-carotene was measured in trial grown only at New Delhi. To estimate kernel β-carotene, three self-pollinated cobs in each of the genotypes were used, while open-pollinated cobs were used for recording grain yield and other agronomic traits. Grain yield (tonnes/hectare) was calculated considering fresh weight per plot, dry matter, shelling percent and moisture at 15 percent.

### Biochemical analysis for estimation of β-carotene

Carotenoid compounds are sensitive to light, heat, and oxygen. Hence self-fertilized cobs from the selected genotypes were individually harvested with husk and the bulked seeds of each genotype were stored in dark at 4°C. The extraction of carotenoids from kernels was carried out using the procedure described by Kurilich and Juvick [28]. β-carotene was estimated using a Water Alliance HPLC system (Waters Chromatography, Milford, Massachusetts, USA). The samples were eluted through YMC Carotenoid C30 column (5 µm, 4.6×250 mm) and detected with a photodiode array detector (PDA). The mobile phase consisted of methanol: tert-butyl methyl ether (80: 20, v/v), and the flow rate was 1 ml min^–1^. Six dilutions of the β-carotene standard (Sigma Chemicals, St. Louis, USA) were used for making the standard curve for β-carotene, and the concentration of β-carotene in each line was measured by standard regression with external standard. To maximize the detection of β-carotene, absorbance was measured at 450 nm.

### Estimation of endosperm modification and tryptophan in protein

BC_2_F_3_ seeds of the introgressed progenies (VQL1-, VQL2- and HKI161- based) and selfed seeds of reconstituted hybrids (Vivek QPM-9 -based) having favourable alleles of *opaque2* and *crtRB1*, along with their original versions were used for the estimation of endosperm modification and tryptophan in protein. Endosperm modification was measured through standard light box test [29], and tryptophan in the endosperm flour was estimated using papain based colorimetric method [30]. The endosperm protein was measured by Micro-Kjeldahl Procedure [31], and was used for computing percent tryptophan in endosperm protein.

## Results

### Marker polymorphism

**Figure 3:**
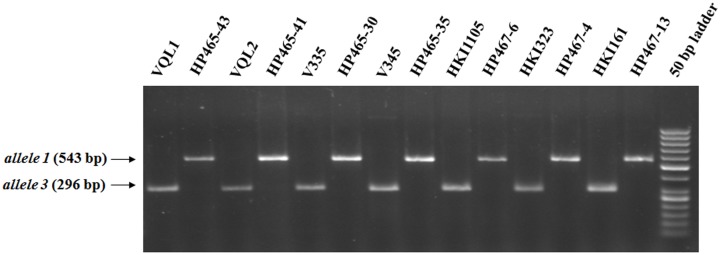
Recurrent and donor inbreds showing polymorphism for alleles of *crtRB1* gene.

The search for recurrent and donor parent polymorphism for *crtRB1* showed a 543 bp amplicon (favourable allele; *allele 1*) in the donor parent whereas a distinct 296 bp amplicon and a faint 1221 bp amplicon were generated in the recurrent parent (Fig. 3). Since the 296 bp amplicon was distinct and clearly polymorphic with *allele1* (543 bp) of the donor parent, the amplicon in the recurrent parent was referred to as *allele 3* (Fig. 3). Of the 200 genome-wide SSR markers screened between the recipient and donor parents, the number of polymorphic markers ranged from 66 (33%) in HKI161×HP467-13 to 82 (41%) in V345×HP465-35 (Table 2). The number and distribution of cross-specific polymorphic markers is presented in Table 2. These polymorphic markers identified were used for recovering the respective RPG.

**Table 2:** Distribution of cross-specific polymorphic SSR markers identified and used for background selection.

Chromosome	Totalmarkers	Polymorphic markers						
		VQL1×HP465-43	VQL2×HP465-41	V335×HP465-30	V345×HP465-35	HKI1105×HP467-6	HKI323×HP467-4	HKI161×HP467-13
1	20	7	7	10	12	7	9	7
2	20	10	7	6	10	11	9	6
3	38	17	20	18	14	13	18	12
4	17	6	8	5	5	4	7	5
5	13	5	6	6	8	5	6	7
6	26	12	10	14	14	11	9	9
7	22	6	6	9	6	4	6	7
8	10	2	1	2	5	4	3	3
9	16	4	3	6	5	4	8	5
10	18	6	4	3	3	5	5	5
Total	200	75	72	79	82	68	80	66
Percentage(%)		37.5	36	39.5	41	34	40	33

### Marker-assisted introgression of allele 1

#### a) BC_1_F_1_ generation

Foreground selection using a marker specific to the *crtRB1* gene in BC_1_F_1_ population resulted in identification of five heterozygous plants (*allele 1*/*allele* 3) in VQL2×HP465-41 to 18 heterozygous plants (*allele 1*/*allele* 3) in V345×HP465-35 (Table 3). Chi-square test showed four (VQL1×HP465-43, VQL2×HP465-41, V335×HP465-30, and HKI323×HP467-4) of the seven crosses deviating from the expected Mendelian segregation pattern (Table 3). The recovery of RPG varied from 70.3% to 88.4% across seven crosses.

**Table 3:** Segregation pattern of alleles of the *crtRB1* gene in different backcross- and selfed- generations across seven crosses.

Cross	Generation	Populationsize	No. ofhomozygotes(*allele1/allele1)*	No. ofheterozygotes(*allele1/allele3*)	No. ofhomozygotes(*allele3/allele3*)	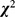	*P* value
VQL1×HP465-43	BC_1_F_1_	59	-	11	48	23.20	0.0001*
	BC_2_F_1_	81	-	16	65	29.64	0.0001*
	BC_2_F_2_	121	31	43	47	14.35	0.0008*
VQL2×HP465-41	BC_1_F_1_	22	-	5	17	6.54	0.0105*
	BC_1_F_2_	30	8	15	7	0.06	0.9672
V335×HP465-30	BC_1_F_1_	38	-	8	30	12.73	0.0004*
	BC_2_F_1_	39	-	13	26	4.33	0.0374*
	BC_2_F_2_	92	19	44	29	2.34	0.3092
V345×HP465-35	BC_1_F_1_	40	-	18	22	0.40	0.5271
	BC_2_F_1_	39	-	14	25	3.10	0.0782
	BC_2_F_2_	60	14	30	16	0.13	0.9355
HKI1105×HP467-6	BC_1_F_1_	37	-	15	22	1.32	0.2498
	BC_2_F_1_	48	-	17	31	4.08	0.0433*
	BC_2_F_2_	88	18	36	34	8.72	0.0127*
HKI323×HP467-4	BC_1_F_1_	38	-	9	29	10.52	0.0012*
	BC_2_F_1_	35	-	10	25	6.42	0.0112*
	BC_2_F_2_	90	19	38	33	6.53	0.0381*
HKI161×HP467-13	BC_1_F_1_	20	-	8	12	0.80	0.3711
	BC_2_F_1_	45	-	10	35	13.88	0.0002*
	BC_2_F_2_	87	23	34	30	5.27	0.0715

*significant at *P*<0.05.

#### b) BC_2_F_1_ generation

Ten heterozygous (*allele 1*/*allele* 3) plants in HKI323×HP467-4 to 17 heterozygous plants (*allele 1*/*allele* 3) were identified in HKI1105×HP467-6 (Table 3). Chi-square test showed five crosses (VQL1×HP465-43, V335×HP465-30, HKI1105×HP467-6, HKI323×HP467-4, and HKI161×HP467-13) deviating from the expected segregation pattern (Table 3). Background selection in the heterozygous plants using polymorphic SSRs led to the recovery of 86.4% RPG in V345×HP465-35 to 93.7% RPG in VQL1×HP465-43. Selfing of the selected heterozygotes led to BC_2_F_2_ generation.

#### c) BC_2_F_2_ generation and BC_1_F_2_ generation

**Figure 4:**
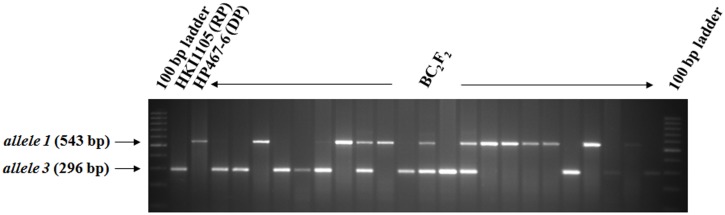
Segregation of *allele1* and *allele 3* in BC_2_F_2_ generation (HKI1105×HP467-6) using the *crtRB1* gene specific marker (RP: Recurrent Parent; DP: Donor Parent).

**Figure 5:**
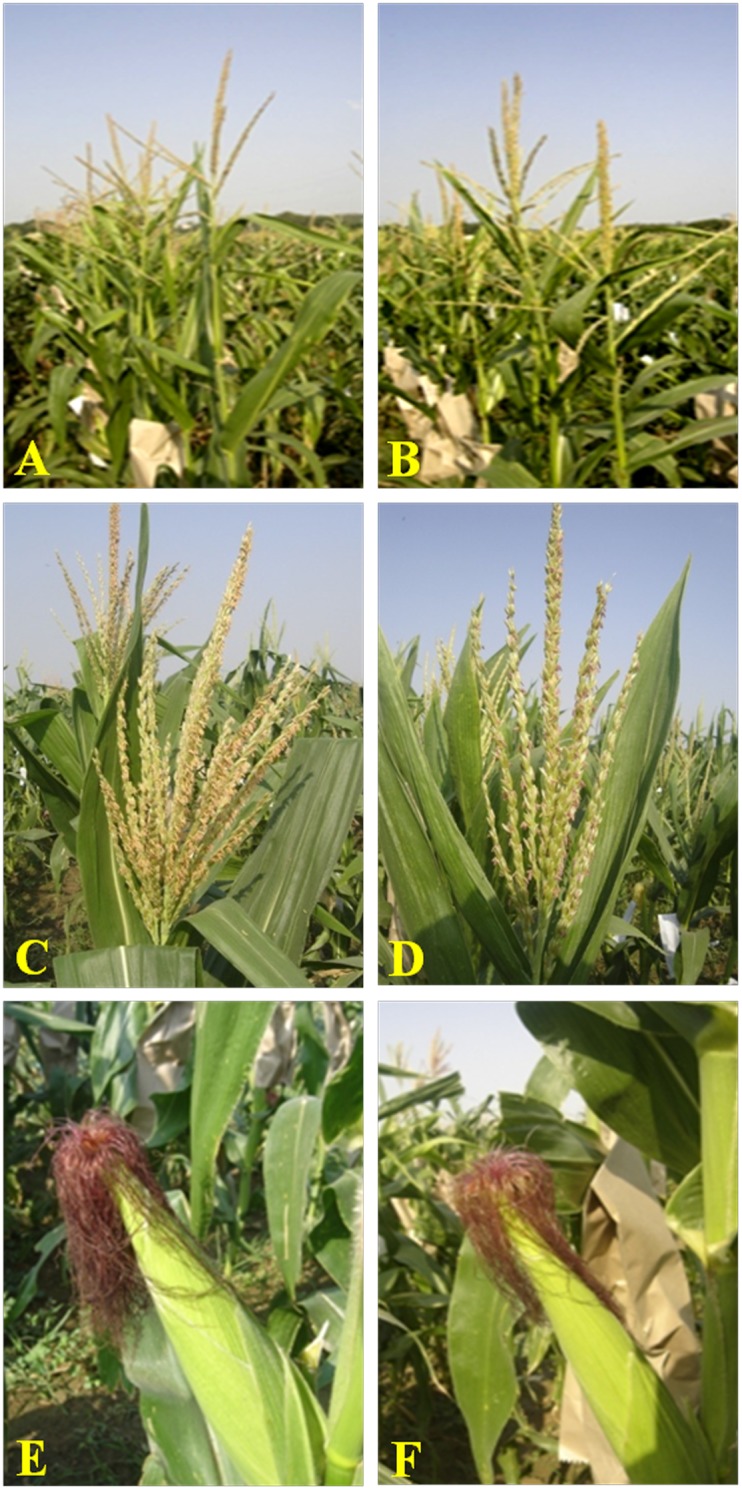
Plant-, tassel- and ear- characteristics of original and β-carotene rich versions of the parental inbreds. **A**: VQL1; **B**: Improved VQL1; **C**: HKI1105; **D**: Improved HKI1105; **E**: HKI161; **F**: Improved HKI161.

A total of 538 BC_2_F_2_ plants across six crosses (except VQL2×HP465-41) were raised and subjected to foreground selection for identifying homozygous plants (*allele 1*/*allele 1*) (Fig. 4). Foreground selection helped in identification of 31 homozygous plants for *allele 1* in VQL1×HP465-43 and 14 plants in V345×HP465-35 (Table 3). Three crosses, viz. VQL1×HP465-43, HKI1105×HP467-6 and HKI323×HP467-4, deviated from the expected segregation pattern of 1∶2∶1 in the BC_2_F_2_ generation (Table 3). Thirty BC_1_F_2_ individuals were raised for VQL2×HP465-41, and foreground selection identified eight homozygous plants (*allele 1*/*allele 1*). The segregation pattern corresponded to the expected Mendelian ratio of 1∶2∶1 (Table 3). The selected homozygotes were self-pollinated to generate BC_2_F_3_/BC_1_F_3_ seeds. The phenotypic features of the selected introgressed progenies and their respective original inbreds are presented in Fig. 5.

### Marker-assisted selection for *opaque2* allele

In the backcross progenies of QPM based crosses, selection for *o2* allele was also carried out among the *crtRB1*-positive heterozygous plants (BC_1_F_1_ and BC_2_F_1_) and homozygous (BC_2_F_2_) plants. For the VQL2-based family, homozygous plants (*o2*/*o2*) were selected in BC_1_F_1_. Thus no further selection for the *o2* allele was required in the advanced generations as the desirable allele had already been fixed. In case of VQL1- and HKI161-based families, heterozygous plants (*O2*/*o2*) were selected and advanced in both BC_1_F_1_ and BC_2_F_1_ generations. Six and nine plants were found to be double homozygotes (*allele1/allele1/o2/o2*) in VQL1- and HKI161-based BC_2_F_2_ families, respectively.

### Kernel quality attributes in selected introgressed inbreds

Kernel β-carotene was estimated in BC_2_F_3_ seeds of three randomly selected introgressed inbreds of each of six crosses while BC_1_F_3_ seeds were evaluated for VQL2-based introgressed inbreds. The concentration of β-carotene varied from 6.0 µg/g in the V335-based introgressed inbreds to 14.7 µg/g among the VQL1-based introgressed inbreds. Convinced with profound effect of *crtRB1* favourable allele, further analyses were carried out in BC_2_F_5_/BC_1_F_5_ seeds of selected introgressed inbreds evaluated in a replicated trial. Concentration of kernel β-carotene among the MAS-derived inbreds varied from 8.6 to 17.5 µg/g (Table 4). Introgression of *allele 1* led to a maximum (12.6-fold) increase in kernel β-carotene in progeny of V335×HP465-30 and a minimum (5.7-fold) increase in progeny of V345×HP465-35. The mean kernel β-carotene for all the recurrent parents was 1.4 µg/g, whereas the same was 14.1 µg/g for the introgressed inbreds. Concentration of kernel β-carotene and recovery of RPG of the selected progenies in each of the seven crosses are presented in Table 4.

**Table 4:** Recovery of recurrent parent genome and kernel β-carotene concentration of the MAS-derived parental inbreds used for reconstitution of hybrids.

Hybrid	Genotypes*	Recovery of RecurrentParent Genome (%)**	β-carotene(µg/g)***	Foldchange
Vivek QPM-9 (VQL1×VQL2)	VQL1 (RP)	-	1.4	-
	HP465-43 (DP)	-	17.8	-
	VQL1-K10-17-43-10	93.7	17.5	12.5
	VQL1-K10-40-11-53	93.7	17.1	12.2
	VQL1-K10-40-11-75	93.7	16.4	11.7
	VQL2 (RP)	-	1.3	-
	HP465-41 (DP)	-	16.8	-
	VQL2-K10-08-14	83.1	16.3	12.5
Vivek Hybrid-27 (V335×V345)	V335 (RP)	-	1.3	-
	HP465-30 (DP)	-	16.5	-
	V335-K10-19-13-08	91.8	16.4	12.6
	V345 (RP)	-	1.5	-
	HP465-35 (DP)	-	13.9	-
	V345-K10-15-23-14	86.4	13.4	8.9
	V345-K10-16-02-18	88.1	8.6	5.7
HM-4 (HKI1105×HKI323)	HKI1105 (RP)	-	1.3	-
	HP467-6 (DP)	-	14.6	-
	HKI1105-K10-01-35-13	91.1	13.3	10.2
	HKI1105-K10-01-35-15	91.1	14.1	10.8
	HKI323 (RP)	-	1.5	-
	HP467-4 (DP)	-	11.3	-
	HKI323-K10-22-15-43	88.7	9.2	6.1
	HKI323-K10-22-24-06	89.5	10.1	6.7
HM-8 (HKI1105×HKI161)	HKI161 (RP)	-	1.3	-
	HP467-13 (DP)	-	16.9	-
	HKI161-K10-02-03-04	89.6	16.0	12.3
	HKI161-K10-02-44-08	91.5	15.1	11.6

RP: Recurrent Parent; DP: Donor Parent;

*- BC_2_F_4_ generation except for VQL2, which is based on BC_1_F_4_ generation;

**- based on BC_2_F_1_ generation except for VQL2, which is based on BC_1_F_1_generation;

***- BC_2_F_5_ seeds except for VQL2, which is based on BC_1_F_5_ seeds.

The introgressed QPM progenies (VQL1-, VQL2-, and HKI161- based) revealed similar degree of endosperm modification as compared to their respective original inbreds. VQL1- and VQL2- based progenies showed ∼25% opaqueness, while HKI161- based progenies had ∼50% opaqueness. The average tryptophan in endosperm protein of VQL1-based progenies was 0.53%; the corresponding value in the recurrent parent was 0.54%. The same for VQL2-based progenies was observed to be 0.56%, as compared to 0.58% in the recurrent parent. In the introgressed progenies of HKI161 and the recurrent parent, the proportions were 0.77% and 0.80%, respectively.

### Kernel quality attributes of reconstituted hybrids

Kernel β-carotene concentration in the reconstituted hybrids ranged from 10.5 µg/g in improved version of HM-4 to 21.7 µg/g in improved version of HM-8 (Table 5). The improved versions of Vivek QPM-9, Vivek Hybrid-27 and HM-8 showed kernel β-carotene >15.0 µg/g, whereas improved versions of HM-4 showed 10.5 to 12.5 µg/g, with an increase of ∼5.5 to 6.6-fold over original hybrid. Across the reconstituted hybrids, an average of ∼8.1-fold increase in kernel β-carotene was observed, with a maximum of 10.2-fold increase in VQL1-K10-40-11-53×VQL2-K10-08-14, the improved version of Vivek QPM-9, where the β-carotene concentration increased from 2.1 µg/g in the original hybrid to 21.5 µg/g in the reconstituted hybrid (Table 5).

**Table 5:** Kernel β-carotene concentration and agronomic performance of reconstituted hybrids developed through MAS at Delhi and Dharwad.

Hybrid	β-carotene(µg/g)	Foldchange	Grainyield(t/ha)		100kernelweight(g)		Numberof rows		Cob girth(cm)	
			DEL	DWD	DEL	DWD	DEL	DWD	DEL	DWD
Vivek QPM-9(VQL1×VQL2)	2.1	-	6.1	5.1	23.0	20.2	15.7	15.7	3.9	4.0
VQL1-K10-17-43-10×VQL2-K10-08-14	17.8	8.5	5.6	5.6	22.5	26.2	13.7	14.7	3.6	3.7
VQL1-K10-40-11-53×VQL2-K10-08-14	21.5	10.2	6.0	5.6	26.0	22.3	12.0	14.0	3.6	4.0
VQL1-K10-40-11-75×VQL2-K10-08-14	21.1	10.0	6.0	6.1	25.4	23.8	14.0	14.0	3.7	3.9
Vivek Hybrid-27(V335×V345)	2.0	-	7.1	7.7	29.9	22.8	12.0	13.4	3.9	3.7
V335-K10-19-13-08×V345-K10-15-23-14	15.8	7.9	7.3	7.5	26.4	25.5	12.7	14.4	3.5	4.1
V335-K10-19-13-08×V345-K10-16-02-18	17.0	8.5	7.1	7.4	25.8	26.8	13.4	12.3	3.6	4.1
HM-4 (HKI1105×HKI323)	1.9	-	6.6	6.7	30.9	22.6	13.0	14.4	3.7	3.9
HKI1105-K10-01-35-13×HKI323-K10-22-15-43	10.5	5.5	7.1	7.0	29.3	27.2	12.0	14.7	3.7	4.0
HKI1105-K10-01-35-13×HKI323-K10-22-24-06	12.5	6.6	7.0	6.6	27.0	27.6	11.7	12.0	3.4	4.1
HM-8 (HKI1105×HKI161)	2.6	-	8.4	6.5	32.9	30.0	14.0	14.4	3.9	4.5
HKI1105-K10-01-35-13×HKI161-K10-02-03-04	21.7	8.3	7.9	7.3	29.5	29.5	13.0	12.4	3.6	4.1
HKI1105-K10-01-35-15×HKI161-K10-02-44-08	19.4	7.5	8.0	6.5	34.1	26.3	12.4	12.7	3.7	4.2
SE	1.20	-	0.92	1.57	1.97	3.26	0.42	1.05	0.17	0.25

DEL: Delhi; DWD: Dharwad; g: gram; t/ha: tonnes/hectare.

The proportion of tryptophan in endosperm flour was estimated in the β-carotene enriched versions of QPM hybrid, Vivek QPM-9, possessing *allele1*/*allele1*/*o2*/*o2*. The original and the reconstituted versions of Vivek QPM-9 showed high degree of endosperm modification with ∼25% opaqueness. The average concentration of tryptophan in endosperm protein among the reconstituted versions of Vivek QPM-9 was 0.81%, while that of the original Vivek QPM-9 was 0.83%.

### Agronomic performance of the reconstituted hybrids

**Figure 6:**
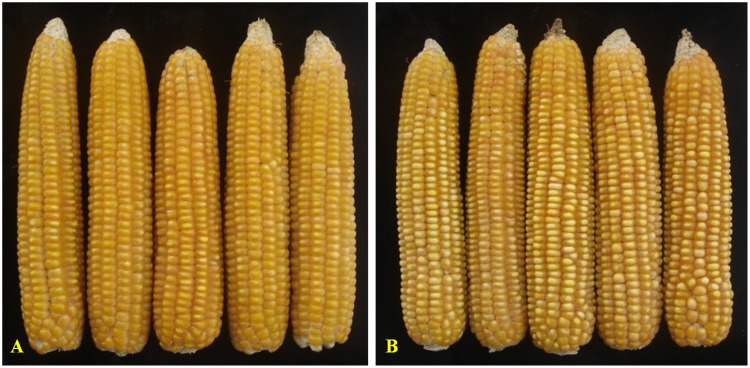
Ear- and grain- characteristics of the original and reconstituted version of hybrid. **A**: Vivek Hybrid-27; **B**: Improved Vivek Hybrid-27.

The data on grain yield and yield-attributing characters of the reconstituted hybrids (generated by crossing the improved versions of their parental lines) are presented in Table 5 and Table 6. In both the locations, β-carotene enriched reconstituted hybrids showed grain yield on par with the respective original hybrids (Table 5, Fig. 6). Vivek QPM-9 produced a grain yield of 6.1 and 5.1 tonnes/hectare at Delhi and Dharwad, respectively, whereas the grain yield of the improved versions was 5.6–6.0 tonnes/hectare at Delhi, and 5.6–6.1 tonnes/hectare at Dharwad. Improved hybrids also exhibited similarity for important morphological characters of the original hybrids like extra early flowering behaviour of Vivek QPM-9 was retained among the β-carotene-rich versions of the reconstituted hybrids (Table 5 and Table 6).

**Table 6:** Agronomic performance of reconstituted hybrids developed through MAS at Delhi and Dharwad.

Hybrid	Coblength(cm)		Earheight(cm)		Plantheight(cm)		Days to50% anthesis		Days to50% silking	
	DEL	DWD	DEL	DWD	DEL	DWD	DEL	DWD	DEL	DWD
Vivek QPM-9(VQL1×VQL2)	17.0	13.2	97.5	73.1	173.0	148.8	45.0	49.5	47.0	52.5
VQL1-K10-17-43-10×VQL2-K10-08-14	17.4	15.4	97.4	78.8	179.2	180.6	45.0	50.5	45.0	53.0
VQL1-K10-40-11-53×VQL2-K10-08-14	15.6	15.1	96.8	87.5	176.2	186.3	45.0	47.5	45.0	51.5
VQL1-K10-40-11-75×VQL2-K10-08-14	17.0	14.5	97.2	78.1	177.8	171.3	45.0	45.0	45.0	48.5
Vivek Hybrid-27(V335×V345)	18.2	16.3	92.3	84.4	175.1	164.4	53.0	52.0	53.0	55.0
V335-K10-19-13-08×V345-K10-15-23-14	21.2	16.3	97.0	78.8	184.5	173.1	46.0	53.5	47.5	56.5
V335-K10-19-13-08×V345-K10-16-02-18	20.7	20.5	91.4	85.1	177.0	178.8	46.0	50.5	47.5	53.0
HM-4 (HKI1105×HKI323)	18.1	17.4	89.0	85.1	156.5	168.1	48.0	56.5	49.0	60.5
HKI1105-K10-01-35-13×HKI323-K10-22-15-43	17.0	16.1	84.6	73.2	156.2	155.0	49.5	56.0	49.5	59.0
HKI1105-K10-01-35-13×HKI323-K10-22-24-06	17.2	15.2	79.3	73.2	154.9	163.1	50.0	55.0	51.0	58.0
HM-8 (HKI1105×HKI161)	20.4	16.4	96.5	83.2	173.9	170.6	48.0	57.5	48.5	61.0
HKI1105-K10-01-35-13×HKI161-K10-02-03-04	19.3	17.5	113.6	92.6	181.4	190.0	49.5	59.5	49.0	63.5
HKI1105-K10-01-35-15×HKI161-K10-02-44-08	17.2	15.9	110.4	97.6	185.2	188.1	47.0	57.0	46.5	60.5
SE	0.98	1.08	5.97	7.60	6.65	14.81	0.29	3.78	0.37	3.91

DEL: Delhi; DWD: Dharwad.

## Discussion

Vitamin A is an important micronutrient that plays vital role in vision, growth and development of humans. VAD, widely prevalent worldwide, affects people predominantly dependent on cereals that are mostly deficient in β-carotene. Maize kernels though show tremendous variation for carotenoids, yet they are inherently deficient in provitamin A. In the present investigation, four popular maize hybrids that were low in β-carotene were targeted for enrichment using accelerated MABB strategy. Distinct marker polymorphism was observed between the respective recurrent- and donor- parents for *allele 1* and *allele 3* of *crtRB1* gene. Among the seven inbreds, three QPM inbreds viz. VQL1, VQL2, and HKI161 also showed polymorphism with their respective donor parents for *umc1066*, the SSR marker specific to *o2* gene. Since both the markers for the *crtRB1* and *o2* genes are located within the target genes, individual plants in the populations could be precisely selected (9, 17, 21].

The segregation pattern of *allele 1* and *allele 3* across the seven crosses (Table 3) showed segregation distortion (SD) in BC_1_F_1,_ BC_2_F_1_, and BC_2_F_2_ generations irrespective of population size and among the two alleles, *allele 1* was under-represented. The reason for occurrence of SD could be the presence of many segregation distortion regions (SDRs) throughout the maize genome; and the location of *crtRB1* coincides with SDR10.2, which might lead to SD of *allele 1* [32]. Comparable SD was reported by Babu et al. [22] while validating the effect of this favourable allele where they found five out of eight populations screened showing SD for *allele 1*. The other possible reasons for SD could be the presence of genes such as gametophytic factors (ga) [33], [34].; or naturally occurring gene mutants like *dek* (defective kernel) and *emb* (embryo-specific mutation) [34]. Further, the genetic background of the target allele also influences SD in different generations, which is evident from the fact that different crosses evaluated in the study showed differential SD pattern in the various backcross- and selfed- generations [22]. For example, VQL1- and HKI323- based progenies showed SD in all the backcross and selfed generations, whereas V345- based progenies did not show SD in any of the generations (Table 3). A majority of the generations that were evaluated in winter season showed SD, which suggests that SD could have been influenced by the environment, a conclusion also reached by Vancetovic (2008) [35]. Frequent occurrence of SD with under-representation of *allele 1* necessitates assaying a large number of segregating individuals to obtain sufficient number of foreground-positive genotypes.

The MABB approach led to the high recovery of RPG among the introgressed progenies. Marker-assisted background selection by SSR loci distributed throughout the genome helped in selecting the foreground positive progenies possessing high RPG [9], [16]. The extent of RPG recovery ranged from 83.1% (BC_1_F_1_ of VQL2×HP465-41) to 93.7% (BC_2_F_1_ of VQL1×HP465-43) (Table 4). The recovery of VQL2-based progenies is less compared to other introgressed progenies as one generation of backcrossing was performed. However, the variable proportion of RPG (86.4 to 93.7%) among BC_2_F_1_ based introgressed progenies is due to fixation of different proportion of recurrent parent alleles among the heterozygous (for *crtRB1*) plants.

Considerable increase in kernel β-carotene among the introgressed inbreds with an average increase of 10.3-fold suggests that introgression of *allele 1* of the *crtRB1* gene alone has a major effect on accumulation of β-carotene in higher concentrations. Substantial increase in concentration of kernel β-carotene was also observed among the reconstituted hybrids over their respective original hybrids. While, kernel β-carotene in the original hybrids ranged from 1.9–2.6 µg/g, the same in the improved versions of Vivek QPM-9, Vivek Hybrid-27 and HM-8 was ∼15–21 µg/g and it was ∼10–12 µg/g in HM-4 versions. Babu et al. [22] also reported similar trend while validating the effect of a favourable allele of the *crtRB1* gene in tropical maize using F_2_ populations. The increase in kernel β-carotene is due to reduction in the transcript expression of the *crtRB1* gene, which decreases the hydroxylation of β-carotene to further carotenoids in the pathway [21], [22]. Significant differences in the accumulation of β-carotene among the introgressed progenies of a specific genetic background were also observed. The selected introgressed progenies derived from V345×HP465-35 had similar recovery of the RPG (∼86–88%) but showed variation in β-carotene (8.6 to 13.4 µg/g). This variation may be due to the differential interaction of the introgressed genome with the target gene and the genetic background of the recurrent parent [16], [36]. This is further supported by the fact that although all the CIMMYT-HarvestPlus donors used in the present investigation possessed the same favourable allele of the *crtRB1* gene, they varied in kernel β-carotene from 11.3 to 17.8 µg/g (Table 4). Besides, the same allele (from donors) after introgression into seven different genetic backgrounds increased the level of kernel β-carotene in the range of 8.6 to17.5 µg/g. Introgressed progenies of all the seven crosses showed kernel β-carotene concentration lower than that of their respective donor parents although it increased many-fold over their recurrent parent (Table 4). This result suggests that apart from a favourable allele of the *crtRB1* gene, other genetic loci or QTLs with minor effects contribute to the increase of kernel β-carotene concentration in the donor parent. Many such QTLs for accumulation of β-carotene and other kernel carotenoids have been reported earlier in maize [37], [38]. It is interesting to note here that kernel β-carotene concentration in some of the reconstituted hybrids was higher as compared to its parental inbreds. This is presumably due to interactions of various loci (contributed by the two parents) affecting the accumulation of kernel β-carotene. Non-additive gene action may also play important role in contributing to higher accumulation of kernel β-carotene in hybrids [26].

A short duration commercial maize hybrid, Vivek Hybrid-9 was biofortified with enhanced tryptophan in the endosperm using marker-assisted introgression of *opaque2* allele, and was released as Vivek QPM-9 for commercial cultivation [9]. Here, Vivek QPM-9 was targeted for enhancing kernel β-carotene; and it was, therefore, important to retain the endosperm quality in the reconstituted hybrids. The results exhibited that endosperm modification and tryptophan in protein among the *o2*-based introgressed progenies and their hybrid versions were comparable to their respective original genotypes. Selection for *o2* allele has led to the retainment of protein quality among the newly developed β-carotene-rich versions [9], [29]. Comparable degree of endosperm modification among the introgressed progenies as compared to their original version is due to accumulation of modifier genes achieved through background selection; and visual selection using light box helped in the final selection of *o2* based genotypes [29]. Thus, the newly developed hybrid provides higher β-carotene along with high tryptophan which is known to be positively correlated with high lysine. Further, newly developed β-carotene-rich maize hybrids possessed grain yield that was on par when compared to their respective original hybrids. Retention of similar grain yield potential in the improved hybrids is due to high recovery of RPG in the parental lines achieved through background selection. However, minor differences in relation to grain yield and associated traits were also observed among the improved versions over their respective original hybrids. This is possibly due to interactions of smaller fraction of donor genome with the recipient genome (in the introgressed inbred lines) [16]. Minor variations observed in grain yield and its related traits for a specific hybrid between locations could be attributed to environmental factors [39], [40].

HarvestPlus, a CGIAR initiative, has set a target of 15 µg/g of β-carotene in maize kernels to help in alleviating VAD in humans (www.harvestplus.org). The traditional maize varieties with yellow kernels contain low amounts of β-carotene ranging from 0.01 to 4.7 µg/g [27], [41]–[44]. Enrichment of carotenoids in maize has been attempted earlier using transgenic approach: over expression of *crtB* and *crtI* from *Erwinia herbicola*, has been reported to accumulate 10 µg/g of β-carotene in Hi-II maize genotype [41]. Zhu et al. [45] and Naqvi et al. [46] further developed transgenic maize lines (with ∼60 µg/g β-carotene) using combination of five genes (*psy1*, *crtI*, *lycb*, *bch* and *crtW*). However, its successful adoption as a cultivar has not been reported so far. In addition, use of transgenic lines depends upon (i) regulatory clearances and (ii) political as well as socio-economic factors [47], [48]. Moreover, at present many countries have not allowed transgenic crops for commercial cultivation. In contrast, the β-carotene rich maize hybrids generated here using naturally available variant of *crtRB1*, are free from such constraints and thus can be readily utilized.

## Conclusions

The present investigation reporting accelerated development of β-carotene enriched maize using marker-assisted breeding strategy holds immense promise as it selects desirable plants precisely and eliminates large scale biochemical estimation in the segregating generations. The introgressed inbreds possessing favourable allele of *crtRB1* can be used as donor for β-carotene enrichment in the biofortification program. Besides, improved hybrids with enormous increase of β-carotene (>15 µg/g) can be directly utilised in alleviating VAD worldwide.
