# Correction: Development of β-Carotene Rich Maize Hybrids through Marker-Assisted Introgression of *β-carotene hydroxylase* Allele

**DOI:** 10.1371/journal.pone.0122130

**Published:** 2015-03-17

**Authors:** The PLOS ONE Staff

There is an error in Fig. 1 in the article PDF. The authors have provided a corrected version here.

**Fig 1:**
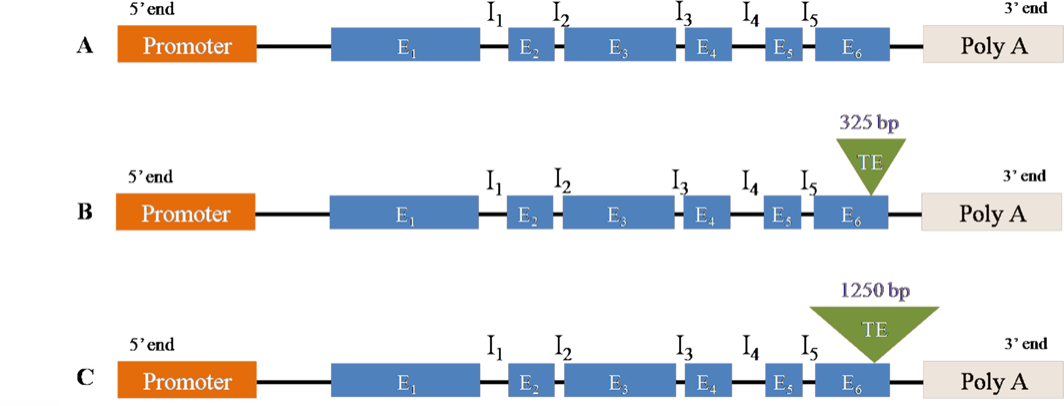
Structure of the alleles of *crtRB1* gene causing variation in β-carotene concentration of maize. E: Exon; I: Intron; TE: Transposable element. A: No TE insertion at 6^th^ Exon causing favourable *allele 1*(543 bp amplicon); B: 325 bp TE insertion at 6^th^ Exon causing unfavourable *allele 2* (296+875 bp amplicon); C: 1250 bp TE insertion at 6^th^ Exon causing unfavourable *allele 3* (296+1221+1880 bp amplicon).
